# Understanding LTP in pain pathways

**DOI:** 10.1186/1744-8069-3-9

**Published:** 2007-04-03

**Authors:** Jürgen Sandkühler

**Affiliations:** 1 Department of Neurophysiology, Center for Brain Research, Medical University of Vienna, Vienna, Austria

## Abstract

Long-term potentiation (LTP) at synapses of nociceptive nerve fibres is a proposed cellular mechanism underlying some forms of hyperalgesia. In this review fundamental properties of LTP in nociceptive pathways are described. The following topics are specifically addressed: A concise definition of LTP is given and a differentiation is made between LTP and "central sensitisation". How to (and how not to) measure and how to induce LTP in pain pathways is specified. The signal transduction pathways leading to LTP at C-fibre synapses are highlighted and means of how to pre-empt and how to reverse LTP are delineated. The potential functional roles of LTP are evaluated at the cellular level and at the behavioural level in experimental animals. Finally, the impact of LTP on the perception of pain in human subjects is discussed.

## Background

Long-term potentiation (LTP) is a much studied cellular model of synaptic plasticity. It is generally defined as the long-lasting but not necessarily irreversible increase in synaptic strength [1,2]. At least two different stages of LTP can be distinguished depending upon its duration and the signal transduction pathways involved. Early phase LTP is independent of *de-novo *protein synthesis and lasts for up to three hours. Late phase LTP involves protein synthesis and lasts longer than three hours, up to the life span of an animal and may involve structural changes at synapses [3]. Short-term potentiation of synaptic strength lasts less than half an hour. Synaptic strength is the magnitude of the post-synaptic response (i.e. the post-synaptic potential or the post-synaptic current, but not action potential firing, see below) in response to a pre-synaptic action potential. LTP can be expressed pre- and/or postsynaptically, i.e. synaptic strength can increase if the release of neurotransmitter(s) is enhanced and/or if the postsynaptic effects of the neurotransmitter(s) become stronger [4]. LTP at synapses in hippocampus is the prime model for learning and memory formation [1]. Recent studies have shown that LTP can also be induced in pain pathways and may contribute to hyperalgesia caused by inflammation, trauma or neuropathy. This review deals with the latter form of LTP.

### What is "central sensitisation"?

"Central sensitisation" is used in the literature in at least two mutually exclusive definitions. Some of the authors use "central sensitisation" as an umbrella term for all forms of changes within the central nervous system which ultimately lead to enhanced pain perception. If using this definition of "central sensitisation" one should keep in mind that none of the presently known phenomena in the central nervous system (CNS) which can be observed in experimental or clinical models of hyperalgesia or allodynia has a proven, causative role for the perception of pain. Thus, all presently proposed mechanisms of "central sensitisation" in this definition would have the status of hypotheses. This includes but is not be limited to the topic of the present review, the LTP at C-fibre synapses in the superficial spinal dorsal horn.

The task force for taxonomy of the International Association for the Study of Pain (IASP) and many other authors define "central sensitisation" as "an enhanced responsiveness of nociceptive neurons in the CNS to their normal afferent input". This is a very clear-cut definition with little error-proneness. The mechanisms underlying "central sensitisation" in this definition can be well studied experimentally. Nociceptive neurons in the CNS may, however, serve very distinct and also antagonistic functions not all of which are related to the perception of pain. Some nociceptive neurons are excitatory, others are inhibitory. Spinal nociceptive neurons may project to different areas in the brain, with a wide spectrum of functions. Other nociceptive spinal neurons may project segmentally to motoneurons and still other may be interneurons with no known function at all. A remarkable exception are those nociceptive neurons which are located in lamina I of the spinal dorsal horn and which express the NK1 receptor for substance P, many of which project supraspinally. If these neurons are destroyed selectively by a toxin conjugated to substance P, then neither inflammation nor neuropathy leads to full expression of hyperalgesia [5,6]. Interestingly, animal responses to acute noxious stimuli are not altered [5,6]. Thus, these neurons are indispensable for the development of hyperalgesia but not essential for acute pain. Enhanced responsiveness of different nociceptive neurons in the CNS may thus have distinct and perhaps opposing consequences on pain. Thus, "central sensitisation" defined in this way is fundamentally different from the former definition. Some forms of "central sensitisation" as defined by the IASP task force could contribute to hyperalgesia and/or allodynia while other forms may rather lead to the contrary, i.e. stronger feedback inhibition and endogenous pain control. Many of the presently known forms of "central sensitisation" defined in the latter way would be assigned a yet unknown (if any) role for pain perception.

An utter confusion is created if the term "central sensitisation" is used but not defined or when switching from one definition to the other within the same publication, i.e. if both definitions are not clearly distinguished. This would incorrectly imply that any form of enhanced responsiveness of nociceptive neurons in the CNS to their normal afferent input would lead to an enhanced perception of pain. Before we evaluate the evidence suggesting that a prolonged increase in synaptic strength at C-fibres contributes to hyperalgesia we will first review the fundamental properties of LTP in pain pathways.

### How to (and how not to) measure LTP

LTP is measured as an increase in mono-synaptically-evoked post-synaptic currents or potentials in response to a single pre-synaptic action potential. LTP is often studied in *in vitro *preparations which allow reliable recordings of synaptic strength. Whole-cell patch-clamp recording is now the most often used technique. It enables some control over the composition of the intracellular fluid of the post-synaptic neurons which may be advantageous to study post-synaptic mechanisms of LTP. If, however a diffusible mediator is involved and dialysis of the post-synaptic neuron has to be avoided, perforated patch-clamp recordings or intracellular recordings with sharp electrodes can be used. To evaluate LTP at the first synapses in nociceptive pathways, transverse slices with long dorsal roots attached can be prepared from lumbar spinal cord of rats or mice to study mono-synaptic, Aδ-fibre or C-fibre evoked excitatory postsynaptic potentials or currents in identified dorsal horn neurons [7,8].

Some aspects of LTP can only be studied in the entire animal with primary afferent nerve fibres and descending pathways from the brain intact. *In vivo *C-fibre-evoked field potentials can be measured in superficial spinal dorsal horn e.g. in response to high intensity electrical stimulation of the sciatic nerve for up to 24 h [9]. These extracellulary recorded field potentials reflect summation of post-synaptic, mainly mono-synaptically-evoked currents but not action potential firing [9,10].

Monitoring presynaptic activity at synapses of primary afferent nerve fibres is technically quite demanding. In an attempt to monitor pre-synaptic activity in primary afferents optical recordings techniques have been utilised. Some voltage-sensitive dyes can be anterogradely transported in primary afferents to the central terminals mainly in lamina I [11] and may serve as an indicator for pre-synaptic electrical activity but not for transmitter release.

LTP can not be directly investigated by recording action potential discharges of post-synaptic neurons, as action potential firing not only depends upon synaptic strength but also on membrane excitability and the balance between excitatory and inhibitory input to the neuron. For the same reasons poly-synaptically-evoked responses can generally not be used to study synaptic strength and changes thereof.

### How can LTP be induced in pain pathways?

#### LTP induction by high frequency electrical nerve stimulation

The most frequently used form of conditioning stimulation to induce LTP at synapses in the brain consists of high frequency electrical stimulation (HFS, around 100 Hz) of an input pathway. Likewise, LTP can be induced at spinal synapses of small diameter primary afferents by conditioning high intensity, high frequency burst-like stimulation (typically 100 Hz bursts given several times for 1 s at C-fibre strength) both, *in vitro *and *in vivo*. In spinal cord slice preparations, both, Aδ-fibre [7] and C-fibre [8,12]-evoked responses are potentiated by HFS when post-synaptic neurons are mildly depolarized to -70 to -50 mV. The same HFS induces, however, long-term depression (LTD) of Aδ-fibre-evoked responses if cells are hyperpolarized to -85 mV suggesting that the polarity of synaptic plasticity is voltage-dependent [7].

**Figure 1:**
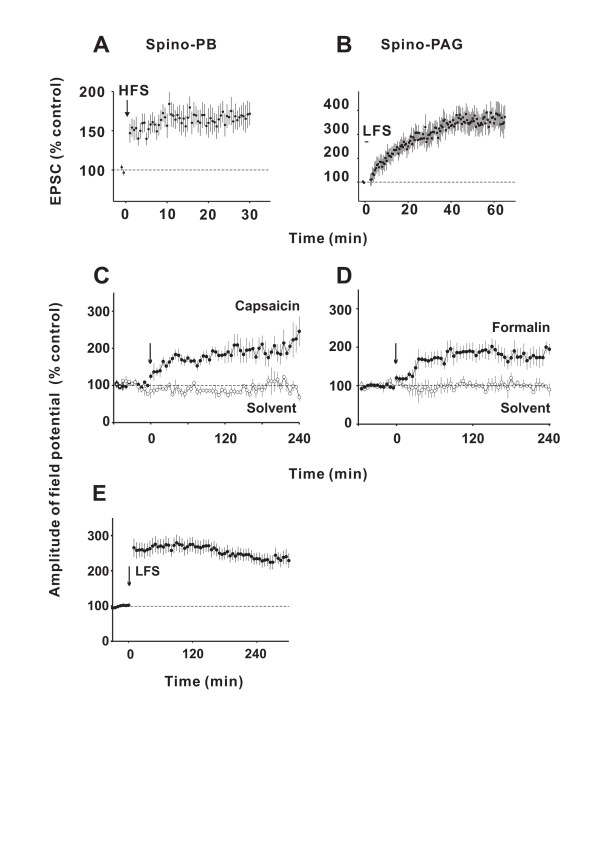
**A, B **show contrasting forms of LTP expressed in distinct groups of spinal lamina I projection neurons *in vitro*. Time courses of mean amplitudes (± SEM) of C-fibre-evoked EPSCs in lamina I neurons with a projection to the parabrachial area (PB, n = 8) or the periaqueductal grey (PAG, n = 7). Conditioning HFS induced LTP in all spino-PB neurons tested (**A**) but was ineffective in spino-PAG neurons (data not shown). Conditioning LFS induced LTP in all 18 spino-PAG neurons tested (**B**) but was never effective in seven spino-PB neurons (data not shown). **C-E **LTP can be induced by natural, low frequency afferent barrage evoked by inflammation of peripheral tissue *in vivo*. Mean time courses of C-fibre-evoked field potentials recorded extracellulary in superficial spinal dorsal horn in response to electrical stimulation of left sciatic nerve of deeply anaesthetized adult rats with spinal cords and afferent nerves intact. Subcutaneous injections of transient receptor potential vanilloid 1 channel agonist capsaicin (1%, 100 μl, n = 5, **C**) or formalin (5%, 100 μl, n = 6, **D**) into the glabrous skin at the ipsilateral hind paw, within the innervation territory of the sciatic nerve at time zero (arrows) induced LTP (closed circles), while injections of the respective solvents (open circles) had no effects (n = 3 in each group). Conditioning electrical LFS (2 Hz, 2 min at C-fibre intensity) of sciatic nerve at time zero (arrow) induced LTP (n = 28, **E**) which was prevented by NMDA receptor antagonist MK-801 (3 mg kg^-1^, i.v.-infusion over 30 min, data not shown). Modified from [12].

Neurons in spinal cord lamina I which express the NK1 receptor play a pivotal role for hyperalgesia in behaving animals [5,6]. Most of these neurons send a projection to supraspinal areas. Interestingly, HFS induces LTP selectively at C-fibre synapses with lamina I neurons which express the NK1 receptor and send a projection to the parabrachial area (Fig. 1). In contrast, HFS fails to induce LTP at synapses with neurons which express the NK1 receptor and send a projection to the periaqueductal grey or at synapses with neurons which do not express the NK1 receptor and which have no identified supraspinal projection [8,12].

HFS at C-fibre intensity of sciatic nerve fibre afferents induces LTP of C-fibre, but not Aβ-fibre-evoked field potentials in superficial spinal dorsal horn of adult, deeply anaesthetized rats [9,13,14]. In contrast, conditioning HFS at A-fibre intensity fails to induce LTP of either A- or C-fibre-evoked field potentials in intact animals. In spinalised animals, conditioning HFS at Aδ-fibre intensity induces, however, LTP of C-fibre-evoked field potentials [15].

#### LTP induced by low frequency electrical nerve stimulation

For most of the C-fibre afferents it is not typical to discharge at rates as high as 100 imp. s^-1^. Some C-fibres may, however, discharge at these high rates but only for short periods of time, e.g. at the beginning of a noxious mechanical stimulus [16]. Many C-fibres discharge at considerably lower rates, around 1–10 imp. s^-1^, e.g. in response to an inflammation or an injury [17]. Conditioning stimulation within this lower frequency band is successfully used to induce LTP at C-fibre synapses. In a spinal cord-dorsal root slice preparation conditioning electrical low frequency stimulation (LFS 2 Hz for 2–3 min, C-fibre strength) of dorsal root afferents induces LTP selectively at C-fibre synapses with lamina I neurons that express the NK1 receptor and project to the periaqueductal grey (Fig. 1) [12]. C-fibre synapses with lamina I neurons which express the NK1 receptor and project to the parabrachial area or with no identified supraspinal projection are, in contrast, not potentiated by LFS [12]. Thus, the pattern and the frequency of discharges in C-fibres determine which synapses at the origin of different ascending pain pathways are potentiated.

Pairing strong post-synaptic depolarization (to +30 mV) with low frequency (2 Hz) stimulation of afferent nerve fibres also leads to a robust LTP in superficial spinal dorsal horn neurons *in vitro *[18]. In spinal cord slices from neonatal rats field potentials evoked by electrical stimulation in the tract of Lissauer are potentiated by repetitive burst-like stimulation at 10 Hz [19].

In deeply anaesthetized adult rats with their spinal cords left intact LFS (at 2 Hz for 2–3 min) of sciatic nerve fibres at C-fibre intensity but not at Aδ-fibre intensity also triggers LTP of C-fibre-evoked potentials (Fig. 1) [12].

In conclusion, HFS and LFS may have fundamentally different effects on LTP induction at different C-fibre synapses. This finding is in line with previous reports also illustrating that the frequency of afferent barrage in C-fibres may have qualitatively different effects in spinal cord. For example, brain-derived neurotrophic factor is released from primary afferents in spinal cord slices in an activity-dependent manner by HFS at 100 Hz but not by 1 Hz LFS of primary afferent nerve fibres [20]. Furthermore, in spinal cord slices from mice HFS (100 Hz) of primary afferent nerve fibres at C-fibre intensity, but not LFS (900 pulses at 1 Hz) selectively induces phosphorylation of extracellular receptor-activated MAP Kinases (ERK1/2) in spinal dorsal horn lamina I [21].

#### Natural noxious stimulation induces LTP in pain pathways

At synapses in the brain LTP induction requires synchronous, high-frequency pre-synaptic activity or pairing of low-level pre-synaptic activity with strong post-synaptic depolarization. At least some of the C-fibre synapses are apparently unique in that LTP can be induced by LFS and by natural, low or high frequency, asynchronous and irregular discharge patterns in sensory nerve fibres. In animals with spinal cord and descending pathways intact, intraplantar, s.c. injections of capsaicin (100 μl, 1%) or formalin (100 μl, 5%) induce slowly rising LTP (Fig. 1) [12].

Some forms of low level afferent input can induce LTP only if descending, presumably inhibitory pathways are interrupted or weakened. Noxious radiant heating of the skin at a hind paw induces LTP in spinalised animals but not in animals with spinal cord intact [22]. Likewise, repetitive, noxious squeezing of the skin or the sciatic nerve induces LTP of C-fibre-evoked field potentials in spinalised rats only [22]. These findings indicate that endogenous antinociceptive systems not only raise thresholds for nociception but also those for the induction of LTP.

#### Pharmacological induction of LTP

At C-fibre synapses LTP can also be induced in the absence of any pre-synaptic activity. Spinal application of a dopamine 1/dopamine 5 receptor agonist (SKF 38393) *in vivo *induces a slowly developing LTP of C-fibre-evoked field potentials which lasts for at least 10 h and which is blocked by the dopamine 1/dopamine 5 antagonist SCH 23390 [23]. In spinalised, deeply anaesthetized, adult rats, superfusions of spinal cord segments with NMDA (10 μM), substance P (10 μM) or neurokinin A (1 μM) are all sufficient to induce LTP of C-fibre-evoked field potentials [24]. With spinal cord and descending (inhibitory) pathways intact spinal applications of NMDA (1–100 μM), substance P (1–100 μM) or neurokinin A (1 or 10 μM) fail, however, to induce LTP of C-fibre-evoked field potentials [24].

### LTP of A-fibre-evoked responses

A-fibre-evoked spinal field potentials are depressed by conditioning 50 Hz stimulation of sciatic nerve fibres. After GABA_A _receptor antagonist bicuculline (1 mg/kg i.p.) the same conditioning stimulus now produces LTP rather than LTD [25]. Similarly, 50 Hz conditioning stimulation produces short lasting potentiation followed by LTD in control animals but LTP in animals with a CCI of sciatic nerve [26]. Topical application of muscimol (10 μg), a GABA_A _receptor agonist to spinal cord prevents tetanus-induced LTP of A-fibre-evoked field potentials in animals with a CCI [27]. This again suggests that the polarity of synaptic plasticity is context-sensitive and not solely dominated by the type of afferent input.

### Signal transduction pathways leading to LTP at C-fibre synapses

**Figure 2:**
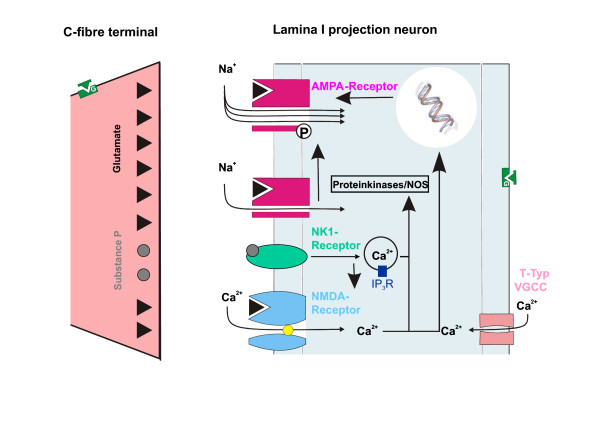
Potential mechanisms of potentiation, prevention and de-potentiation at synapses between C-fibres and spinal cord projection neurons. Conditioning electrical nerve stimulation or natural noxious stimulation triggers release of glutamate and substance P which causes opening of NMDA receptor channels and T-type voltage-gated Ca^2+ ^channel and Ca^2+ ^release from intracellular stores. This activates Ca^2+^-dependent signal transduction pathways including protein kinases and transcription factors. Synaptic strength is probably increased by phosphorylation of synaptic proteins including AMPA receptor channels [55], altered trafficking of synaptic proteins, e.g. increased insertion of AMPA receptors into the sub-synaptic membrane [56] and de-novo protein synthesis. According to this model, LTP can be prevented if release of glutamate and/or substance P is inhibited, for example by activation of pre-synaptic, G-protein-coupled μ-opioid receptors, or if opening of voltage sensitive and Ca2+ permeable ion channels is blocked, e.g. via postsynaptic inhibition by an opioid. Depotentiation could result from de-phosphorylation of synaptic proteins, changes in receptors trafficking and degradation of synaptic proteins.

In principle, LTP can be induced and/or expressed by pre-synaptic [28] or by post-synaptic [29,30] mechanisms or by any combination thereof. At present, there is clear evidence for a post-synaptic, Ca^2+^-dependent form of LTP induction in spinal cord lamina I neurons. Induction of LTP at C-fibre-synapses requires co-activation of NK1 and NK2 receptors [9], opening of ionotropic glutamate receptors of the NMDA type [8,12,13], opening of T-type voltage-gated calcium channels [8,12], and activation of group I but not group II or III metabotropic glutamate receptors [31]. Activation of NK1 receptors by substance P may directly enhance single NMDA channel opening [32] and NMDA receptor mediated currents in lamina I neurons [8] and all this may lead to substantial rise in post-synaptic [Ca^2+^]_i _(Fig. 2). It is presently unknown if Ca^2+ ^influx through Ca^2+^-permeable a-amino-3-hydroxy-5-methyl-4-isoxazolepropionic acid (AMPA) receptors is required for LTP induction in pain pathways. Some indirect evidence suggests, however, that this might be the case [21,33].

In any case, a rise in post-synaptic [Ca^2+^]_i _is essential for LTP induction and the magnitude in [Ca^2+^]_i _rise is linearly correlated with the magnitude of LTP *in vitro *[8]. Recent data demonstrate that LTP-inducing stimuli cause substantial rise in [Ca^2+^]_i _in lamina I neurons not only in slice preparations, but also in intact animals [12]. Not surprisingly therefore signal transduction involves Ca^2+^-dependent pathways including activation of protein kinase C, calcium-calmodulin-dependent protein kinase II (CaMKII), protein kinase A (PKA) phospholipase C (PLC), inositoltriphosphate-3 (IP_3_) receptors, nitric oxide synthase (NOS) and members of the mitogen-activated protein kinase family (MAPK), including extracellular signal-regulated kinase (ERK) (Fig. 2) [8,12,18,34-36].

When assessed with voltage-sensitive dyes the pre-synaptic facilitation of electrical activity in primary afferents after LTP-inducing stimuli is partially sensitive to iNOS inhibitor (AMT), a blocker of glial cell metabolisms (MFA), and an mGluR group I antagonist (LY367385) [11].

Inhibition of protein synthesis in spinal cord by either cycloheximide or anisomycin selectively inhibits the maintenance of the late-phase of spinal LTP but does not affect either LTP induction or baseline responses of C-fibre evoked field potentials [37].

Importantly, the very same signal transduction pathways are required for full expression of hyperalgesia in animal models of inflammatory and neuropathic pain e.g. [38-41].

### How to pre-empt LTP induction in pain pathways

LTP induction can be prevented by blockade of any of the above mentioned essential elements of signal transduction for LTP. In mature rats deep (surgical) level of anaesthesia with either urethane, isoflurane or sevoflurane is, however, insufficient to pre-empt LTP induction of C-fibre-evoked field potentials [42]. LTP is prevented by low dose intravenous infusion of μ-opioid receptor agonist fentanyl [42]. Similarly, LTP of spinal field potentials elicited by stimulation in the tract of Lissauer in spinal cord slices is blocked by DAMGO, a more specific agonist at these receptors [19]. Activation of spinal a_2_-adrenoreceptors by clonidine [43] or spinal application of the benzodiazepine diazepam [44] also prevents LTP induction *in vivo*.

Functional blockade of glial cells by i.t. administration of fluorocitrate changes the polarity of HFS induced synaptic plasticity. When HFS is given 1 h, but not 3 h after fluorocitrate LTD but no LTP of C-fibre-evoked field potentials is induced [14].

### LTP can be reversed

LTP of C-fibre-evoked field potentials can be reversed by brief, high frequency conditioning electrical stimulation of sciatic nerve fibres at Aδ-fibre intensity [15]. Reversal of LTP by Aδ-fibre stimulation is time-dependent and effective only when applied 15 or 60 min but not 3 h after LTP induction [45].

Spinal application of either, NK1 or NK2 receptor antagonists one to three hours after HFS, i.e., after LTP is established, does not affect maintenance of LTP [9], suggesting that activation of these receptors, which are required for the induction of LTP are not essential for its maintenance.

### What is the functional role of LTP in pain pathways?

Modulation of synaptic strength is a powerful mechanism to control signal flow in selected pathways. A typical consequence of LTP at excitatory synapses would be an increase in action potential firing of the same and perhaps also of downstream neurons in response to a given stimulus. And indeed, LTP-inducing conditioning stimuli have been found to facilitate action potential firing of multireceptive neurons in deep dorsal horn e.g. [46-48]. This is likely due to LTP at the first synapse in the nociceptive pathway but other mechanisms of facilitation should not be excluded. Action potential firing would also be enhanced if membrane excitability is increased, i.e. the thresholds for action potential firing are lowered, if inhibition is less effective or if inhibition is even reversed and becomes excitatory e.g. due to a reversal of the anion gradient in the post-synaptic neuron [49,50].

HFS of sciatic nerve fibres which induces LTP at synapses of C-fibres in spinal cord has behavioural consequences in rats and causes thermal hyperalgesia at the ipsilateral hind paw for six days [34]. This suggests that LTP at C-fibre synapses has an impact on nociceptive behaviour.

### Perceptual correlates of LTP in pain pathways in human subjects

An indispensable proof for any proposed mechanism of hyperalgesia is an appropriate correlate in the human. And indeed, conditioning HFS of cutaneous peptidergic afferents in humans cause increased pain perception in response to electrical test stimuli applied through the same stimulation electrode [51]. Noxious stimulation with punctate mechanical probes in skin adjacent to the HFS conditioning skin site uncovers a marked (2–3 fold) increase in pain sensitivity, i.e. secondary hyperalgesia [51]. Touching the skin around the conditioning stimulation electrode with a soft cotton wisp evokes pain only after HFS. Thus, HFS also induces secondary mechanical allodynia. Hyperalgesia at the conditioned site but not secondary hyperalgesia or allodynia at adjacent skin areas is prevented by pre-treatment with ketamine [52], a clinically used substance which, among other effects, also blocks NMDA receptors.

Interestingly, all thermal modalities comprising cold and warm detection thresholds, cold and heat pain thresholds as well as pain summation (perceptual "wind-up") remain unaltered after conditioning HFS of peptidergic skin nerve fibres [53].

When verbal pain descriptors are used to evaluate pain in addition to its perceived intensity after HFS, a significant long-term increase in scores for sensory but not for affective descriptors of pain is detected [54]. Within the sensory descriptors, those describing superficial pain, those for heat pain and those for sharp mechanical pain are all potentiated. The authors conclude that brief painful stimuli rarely have a strong affective component and that perceived pain after HFS exhibits predominantly a potentiation of the C-fibre-mediated percepts hot and burning [54].

In humans subjects conditioning LFS causes also an increased pain sensitivity in the area around the LFS conditioned skin site but a depression of pain evoked by stimulation through the same electrode [51].

## Conclusion

LTP at synapses between primary afferent C-fibres and a group of nociceptive neurons in spinal cord lamina I which express the NK1 receptor for substance P is a potential mechanism underlying some forms of pain amplification in behaving animals and perhaps human subjects. Both, LTP and hyperalgesia involve the same essential elements, i.e. primary afferent C-fibres and lamina I neurons which express the NK1 receptor. Further, induction protocols, pharmacological profile and signal transduction pathways are virtually identical.

## Abbreviations

AMPA α-amino-3-hydroxy-5-methyl-4-isoxazolepropionic acid

CaMKII Calcium-calmodulin-dependent protein kinase II

CNS Central nervous system

HFS High frequency stimulation

IASP International Association for the Study of Pain

IP_3 _Inositol 1,4,5-trisphosphate

LFS Low frequency stimulation

LTP Long-term potentiation

MAPK Mitogen-activated protein kinase

NK1 Neurokinin 1

NK2 Neurokinin 2

NMDA N-methyl-D-aspartate

NOS Nitric oxide synthase

PKA Protein kinase A

PKC Protein kinase C

PLC Phospholipase C

## Competing interests

The author declares that he has no competing interests.
